# Efficacy of Venetoclax and Dexamethasone in Refractory IgM Primary Plasma Cell Leukemia with t(11;14) and TP53 Mutation: A Case Report and Literature Review

**DOI:** 10.1155/2020/8823877

**Published:** 2020-12-28

**Authors:** Salimah Valliani, Mir Ali, Omar Mahmoo, Sanjay Hinduja, Calvin K. Chen, Lloyd Damon, Haifaa Abdulhaq

**Affiliations:** ^1^University of California San Francisco-Fresno (UCSF Fresno), Fresno, California, USA; ^2^Community Regional Medical Center (CRMC), Fresno, California, USA; ^3^University of California San Francisco (UCSF), San Francisco, California, USA

## Abstract

Primary plasma cell leukemia (pPCL) is an uncommon disease. IgM multiple myeloma (MM) is an infrequent subtype that accounts for less than 1 percent of MM cases. IgM pPCL is quite rare with only a few cases published to date. We describe a case of a patient with IgM pPCL who initially presented with hyperviscosity syndrome requiring urgent plasma exchange. His bone marrow biopsy demonstrated t(11;14). He progressed on proteasome inhibitors, immunomodulating agents, and other chemotherapy medications but later achieved very good partial response (VGPR) to venetoclax and dexamethasone. Given the poor prognosis of pPCL, further studies using venetoclax alone or in combination with other novel agents as first-line treatment options are warranted particularly in patients with t(11;14).

## 1. Background

Plasma cell leukemia (PCL) is an aggressive variant of multiple myeloma (MM). The diagnostic criteria initially proposed required more than 20% circulating plasma cells and an absolute plasma cell count greater than 2 × 10^9^/L in peripheral blood. The International Myeloma Working Group considers one of the above two criteria to be sufficient for diagnosis [1]. Primary PCL (pPCL) is a terminology used for a patient who meets the above criteria at initial diagnosis with no prior history of MM. pPCL accounts for only 1-2% of cases at initial diagnosis of multiple myeloma [2]. Secondary PCL is a leukemic transformation of relapsed or refractory MM.

IgM-subtype of MM occurs in only 0.8% of MM cases [3]. Waldenstrom's disease is the more common presentation for an IgM monoclonal lymphoproliferative neoplasm. However, patients who have elevated serum IgM monoclonal protein along with features of multiple myeloma including clonal plasma cells in the marrow and lytic bone lesions should be diagnosed as IgM MM [3]. IgM pPCL is a particularly rare entity with only a handful of cases reported. pPCL has a poor prognosis even with the utilization of novel agents. pPCL is known to have a high incidence of cytogenetic and molecular abnormalities such as 17p deletion, TP53 mutation, and 1q gain. t(11;14) is present in 15–20% of MM patients, while reported in up to 50% of patients with pPCL.

Overexpression of BCL-2 and other antiapoptotic proteins is a hallmark of tumor cell survival in MM [4]. Venetoclax, a selective, oral, BCL-2 inhibitor, has shown activity in relapsed and refractory multiple myeloma, specifically in patients with t(11;14) [5]. Few cases have reported the activity of venetoclax in PCL, but none reported in IgM pPCL. We describe a case of IgM pPCL with t(11;14) that demonstrated excellent response to venetoclax and dexamethasone (ven/dex) combination.

## 2. Case Presentation

**Figure 1:**
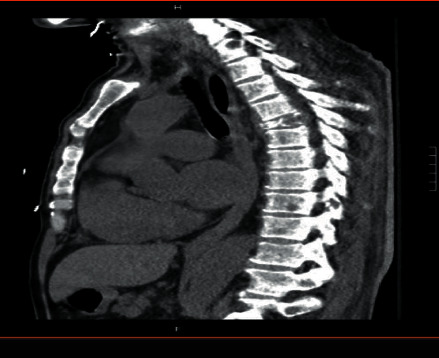
CT scan showing lytic bone lesions.

**Figure 2:**
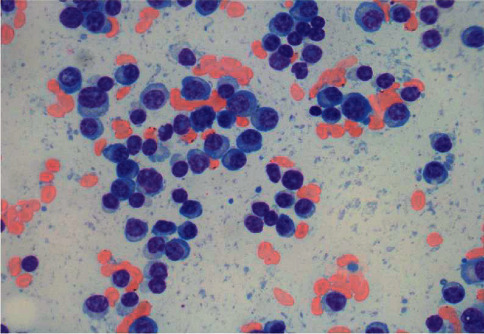
Plasma cells in bone marrow aspirate smear.

**Figure 3:**
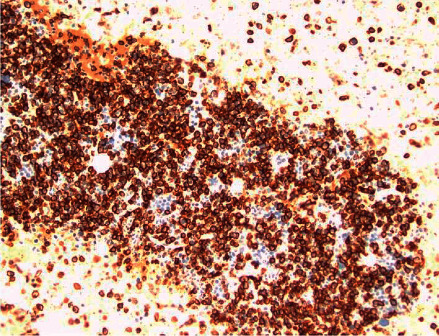
Bone marrow CD 138 IHC stain.

**Figure 4:**
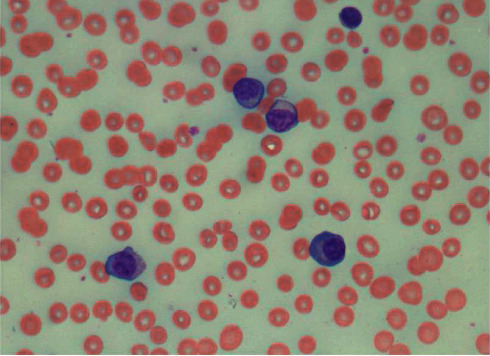
Circulating plasma cells in peripheral blood smear.

A 58‐year‐old man with no known hematologic disease presented with fatigue, lethargy, and increased drowsiness for a few weeks. He also had episodes of confusion, headache, and epistaxis. A review of symptoms was negative for fever, night sweats, or weight loss. His past medical history included Hurthle cell thyroid carcinoma, which was treated with surgery and radioiodine therapy. The patient was initially admitted to an outside hospital for altered mental status. His symptoms were attributed to *E. coli* sepsis and suspected hepatic encephalopathy. He was treated with intravenous antibiotics with partial improvement in symptoms. He was discharged but subsequently readmitted and transferred to our facility within a few days with worsening mental status. He continued to have intermittent epistaxis and hemoptysis. On physical examination, he was noted to be somnolent. Blood crusting was noted on his nose. Initial laboratory data at our institution were as follows: hemoglobin, 8 gm/dl; platelet count, 60,000/*μ*l; and white blood cell count, 15,600/*μ*l. A complete metabolic panel showed creatinine, 0.6 mg/dL; BUN, 13 mg/dl; calcium, 9.2 mg/dl (10.2 when corrected for albumin); total protein, 12.2 g/dl; albumin, 2.6 g/dL; alkaline phosphate, 75 *μ*/l; AST, 8 *μ*/l; ALT, 14 *μ*/l; and total bilirubin, 0.2 mg/dl. Serum viscosity was elevated at 4.22. Serum protein electrophoresis and immunofixation showed an IgM-kappa monoclonal protein with M protein of 7.3 gm/dL and IgM of 10,000 mg/dL. Bone survey and computed tomography scan (CT scan) findings were consistent with multiple lytic lesions in the vertebrae (Figure 1) and hepatosplenomegaly. Magnetic resonance imaging (MRI) brain was unremarkable. Bone marrow biopsy showed >90% kappa-restricted plasma cells, which were CD 138 positive by immunohistochemistry (Figures 2 and 3). Fluorescence in situ hybridization (FISH) was positive for t(11; 14) CCND1/IGH and 13q del. MYD88 mutation by polymerase chain reaction (PCR) analysis was negative. Cytogenetics showed complex karyotype with del [6] (q25), add [1] (p13), del [7] (q13q23), add [8] (q24), del [9] (q22q34), t(11;14) (q13; q32), −13, −16, and add [10] (q13). Next-generation sequencing on bone marrow biopsy by Foundation One Heme showed IGH-CCND1 rearrangement and a TP53 mutation. Peripheral blood flow cytometry demonstrated 54.3% plasmacytoid B-cells/plasma cells with CD 20 positive and partially positive for CD 19 and HLA/DR. Peripheral smear showed 55% plasma cells leading to the diagnosis of IgM pPCL (Figure 4).

**Figure 5:**
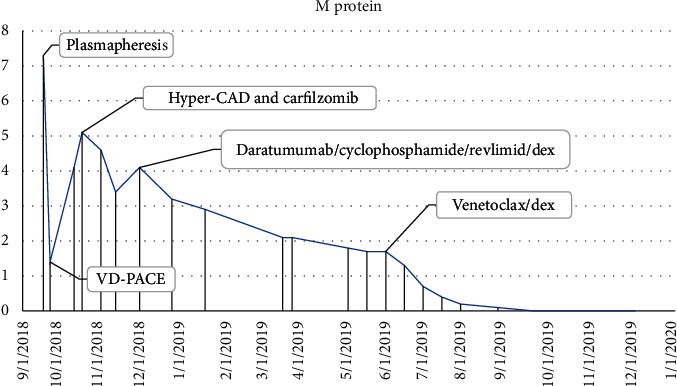
Serum monoclonal proteins.

**Figure 6:**
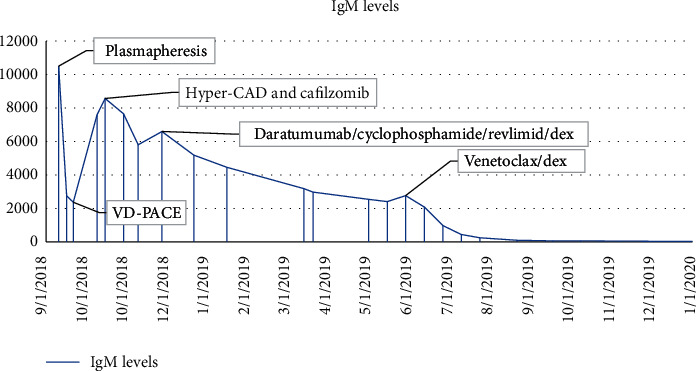
Ig M levels.

The patient was initially treated with daily plasmapheresis for five days, which decreased the IgM levels from >10,000 mg/dl to 2758 mg/dl with significant improvement in his symptoms of headache, confusion, and epistaxis. He was then started on combination chemotherapy with bortezomib, dexamethasone, cisplatin, adriamycin, cyclophosphamide, and etoposide (VD-PACE). Following the first cycle of VD-PACE, IgM increased steeply to 8568 mg/dl. Given the aggressive nature of the disease, treatment was changed to hyper-CAD (fractionated cyclophosphamide, doxorubicin, and dexamethasone) plus carfilzomib. Prior data show that the combination of proteasome inhibitors and hyper-CAD is effective in refractory multiple myeloma cases and can often serve as a bridge to subsequent treatment [7]. IgM level dropped to 5798 mg after one cycle. However, treatment was stopped after one cycle as the patient developed an acute decrease in cardiac ejection fraction. Treatment was changed to daratumumab, lenalidomide, cyclophosphamide, and dexamethasone after two months of initial diagnosis. After three cycles, IgM decreased from 5000 mg/dL to 2500 mg/dL, and bone marrow plasma cells decreased to 30%. With three additional cycles, IgM plateaued at 2400, and a repeat bone marrow biopsy showed an increase of plasma cells to 50%. He was then switched to ven/dex after eight months of initial diagnosis. He received venetoclax at 400 mg daily and dex at 40 mg weekly. He achieved a very good partial response (VGPR) within two months. IgM level decreased to 200 mg/dL, M protein to 0.2 gm/dL, and bone marrow plasma cells to 3%, with no peripheral plasma cells (Figures 5 and 6). The patient was evaluated for autologous hematopoietic cell transplantation (AuHCT) after achieving VGPR. However, mobilization failed despite the use of cyclophosphamide, granulocyte colony-stimulating factor, and plerixafor. The patient continued ven/dex for fifteen months and tolerated treatment well. The only drug-related adverse event has been grade 1 thrombocytopenia. His IgM levels have remained <100 mg/dl with no detectable serum M protein, but immunofixation continues to demonstrate the IgM-kappa monoclonal protein.

## 3. Discussion

Primary plasma cell leukemia has unique pathophysiologic and clinical features that predispose for poor prognosis. pPCL has lower expression of the bone marrow adhesion molecule CD56 expression [11] and downregulation of CXCR4, leading to impaired retention of plasma cells within the bone marrow and increased circulating plasma cells. There is overexpression of CD27 in pPCL, which causes activation of NFkB, leading to higher antiapoptotic activity. There is also a high incidence of molecular and cytogenetic abnormalities, including del 13q, del 17q, 1q gain, TP53 mutation, and hypodiploidy [8]. These features lead to the common findings of cytopenias and increased frequency of extramedullary disease with hepatosplenomegaly, which was noted in our patient. Our patient also had TP53 mutation, which is associated with poor prognosis [9].

In our patient, IgM MM was favored over Waldenstrom macroglobulinemia (WM) due to the presence of bone lytic lesions and t(11;14). Furthermore, the absence of MYD88 mutation favored the diagnosis of IgM MM as this mutation is present in more than 90% of cases of lymphoplasmacytic lymphoma (LPL)/WM [6]. Our patient had symptoms of hyperviscosity with blurred vision and confusion at presentation along with elevated IgM levels. His symptoms improved after plasmapheresis. The overlapping symptoms between LPL/WM and IgM MM can provide a diagnostic challenge. IgM MM is an infrequent subtype that accounts for only 0.8% of MM [3]. As per the Center for International Blood and Marrow Transplant Research (CIBMTR), among 3578 MM patients who received AuHCT over 10 years (1995–2005), only 11 patients had IgM multiple myeloma. This exhibits the rarity of the disease [12]. A retrospective study from the Mayo Clinic database looked at the prognosis of patients diagnosed with IgM MM. Twenty-one patients met the diagnostic criteria of 10% or more plasma cells on bone marrow biopsy, plus the presence of lytic bone lesions and or t(11;14) [3]. These patients had a median overall survival of 30 months, like myeloma population, but shorter than WM. Furthermore, we identified only three cases of IgM PCL reported in the literature. One patient had a durable response to multiagent chemotherapy along with rituximab, but the other two patients responded only briefly before progressing and eventually expiring [13–15]. None of the cases reported using venetoclax as a treatment modality.

Primary PCL has a poor prognosis compared to non-pPCL MM. Based on the SEER database review from 2006–2009, the median overall survival (OS) was around 12 months, even in the era of treatment with novel agents [2]. Primary PCL patients have high early mortality with 15% of deaths reported within a month of diagnosis. They usually present with a higher R-ISS (Revised International Staging system) stage. The treatment goal in primary plasma cell leukemia is focused on the rapid institution of therapy to achieve cytoreduction to reverse and or prevent complications. Treatment options include a combination of novel agents and chemotherapy to achieve a deep response. Retrospective data in pPCL have also shown a clear benefit of consolidation with AuHCT [16].

Few prospective studies evaluated various treatment options in pPCL. In an IFM study [17], bortezomib-based treatment led to improved overall survival (OS) of 36 months and a median progression-free survival (PFS) of 15 months. The overall response rate (ORR) was 72% with 37% response rate for very good partial response (VGPR) + complete response (CR). A GEMIMA study used lenalidomide and dexamethasone, which showed OS of 28 months and PFS of 14 months [18]. Both studies have also demonstrated the benefit of maintenance of bortezomib or lenalidomide, respectively, after AuHCT. Patients who responded to first-line therapy had OS of 26 months compared to 4.2 months in nonresponders. Response criteria in pPCL are MM with the addition of no plasma cells seen in peripheral blood and the absence of extramedullary disease [1]. Our patient had rapid progression on bortezomib- and carfilzomib-based therapies signaling a short survival expectation.

Daratumumab, an anti-CD38 monoclonal antibody, is effective in relapsed refractory MM [19] and should be explored in pPCL. The EMN12 trial of carfilzomib, lenalidomide, and dexamethasone in pPCL showed deep hematologic responses after 4 cycles (≥VGPR in 80% and ≥CR in 33%) [20]. Our patient had a partial response with daratumumab, lenalidomide, and dexamethasone, but this response was not persistent.

Venetoclax is a potent Bcl-2 inhibitor which showed efficacy in relapsed and refractory MM. It has been demonstrated in vitro as well as in vivo that a subgroup of plasma cells is Bcl‐2 dependent and thus sensitive to venetoclax. This subset is restricted to those with t(11;14). It is associated with high Bcl‐2, and low Bcl‐X_L_ and Mcl‐1 mRNA expression, resulting in higher sensitivity to Bcl-2 inhibition [21].

A phase I trial evaluated the response of venetoclax in patients with relapsed refractory MM, the majority of who were refractory to both bortezomib and lenalidomide. The ORR was 21% with VGPR in 15% of patients. ORR and ≥VGPR among patients with t(11;14) group were 40% and 27%, respectively. Median time to progression (TTP) in patients with t(11;14) was 6.6 months compared with 1.9 months in the group without t(11;14) [5]. The randomized phase 3 BELLINI trial showed improvement in PFS with the addition of venetoclax to bortezomib and dexamethasone (22.4 vs 11.5 months), but the trial was suspended due to increased deaths in venetoclax arm. Patients with t(11;14) receiving venetoclax had 95% ORR, including ≥VGPR in 75%, CR or stringent (sCR) in 55%, and minimal residual disease- (MRD-) negativity in 25% versus 47%, 27%, 7%, and 0% with placebo, respectively. There was also a trend towards improvement in OS in patients with t(11;14) [22].

Few case reports described the activity of venetoclax in PCL. In one case, a patient with IgG lambda pPCL with t(11;14) relapsed six months after undergoing an AuHCT. The patient had an excellent response to venetoclax at the dose of 1200 mg daily in 21-day cycles and achieved MRD negativity after nine cycles. He continued to be on treatment at the time of publication [10]. Another case described a patient with lambda light chain pPCL with t(11;14) and TP53 mutation who failed to respond to initial induction treatment with combination chemotherapy consisting of VD-PACE. This patient was treated with a combination of venetoclax, daratumumab, and dexamethasone and achieved a sCR two months after initiating treatment with negative MRD. Venetoclax was dosed at 300 mg daily, and the patient continues to have stringent complete response (sCR) after 10 months of initiation of the treatment [23]. A third case described a patient with kappa light chain-restricted pPCL with del 17p and t(11;14) who had VGPR with carfilzomib, lenalidomide, and dexamethasone and proceeded to AuHCT. She relapsed on day +30 and was treated with bortezomib (days: 1, 4, 8, and 11), venetoclax (800 mg/day), and weekly dexamethasone. After just one cycle, she had dramatic suppression of light chains, and bone marrow evaluation was negative for MRD. The patient continued to be on treatment at the time of publication [24].

Our case highlights the efficacy of venetoclax in pPCL with t(11;14), similar to the above cases but is unique in that it was the rare IgM subtype of pPCL and it was associated with TP53 mutation. The patient achieved a very good partial response with normalization of quantitative IgM protein and disappearance of serum M-protein and plasma cells from peripheral blood. Bone marrow biopsy after two cycles showed only 3% kappa-restricted plasma cells. The patient continues to be on ven/dex after 15 months without significant side effects with IgM level <100 mg/dl showing the durability of response.

This case raises the prospect of evaluating venetoclax as a first-line treatment of pPCL with t(11;14), alone or in combination with other novel agents such as daratumumab and/or lenalidomide. Given the rarity of the disease, it may be challenging to enroll patients in prospective studies to evaluate the above question.

## 4. Conclusions

Our case demonstrates the efficacy of ven/dex in an aggressive and refractory pPCL patient with t(11;14) and TP53 mutation and, in this case, IgM-subtype. Given the poor prognosis of pPCL, further studies incorporating venetoclax in combination with other novel agents in the treatment of this disease particularly in the first-line setting are warranted.
